# Primary Percutaneous Coronary Intervention with High-Bolus Dose Tirofiban: The FASTER (Favorite Approach to Safe and Effective Treatment for Early Reperfusion) Multicenter Registry

**DOI:** 10.1155/2022/9609970

**Published:** 2022-03-29

**Authors:** Stefano Rigattieri, Corrado Lettieri, Gianluca Tiberti, Michele Romano, Marco Ferlini, Luca Testa, Simona Pierini, Federica Ettori, Enrico Passamonti, Alfredo Marchese, Giuseppe Musumeci, Giovanni Esposito, Giuseppe Tarantini

**Affiliations:** ^1^Interventional Cardiology, Sant'Andrea Hospital, Sapienza University, Rome, Italy; ^2^Cardiology Department, Carlo Poma Hospital, Mantua, ASST Mantova, Italy; ^3^Cardiovascular Department, Alessandro Manzoni Hospital, Lecco, ASST Lecco, Italy; ^4^Cardiology Department, Fondazione IRCCS Policlinico San Matteo, Pavia, Italy; ^5^Department of Cardiology, IRCCS Policlinico San Donato, Milan, Italy; ^6^Ospedale Bassini, Cinisello Balsamo, Italy; ^7^Cardiology Unit, ASST Degli Spedali Civili di Brescia, Brescia, Italy; ^8^Ospedale di Cremona, Struttura Complessa di Cardiologia, Cremona, Italy; ^9^Interventional Cardiology, Santa Maria Hospital, Bari, Italy; ^10^Cardiology Department, A. O. Ordine Mauriziano di Torino, Turin, Italy; ^11^Department of Advanced Biomedical Sciences, University of Naples Federico II, Naples, Italy; ^12^Department of Cardiac, Thoracic, Vascular Sciences and Public Health, University of Padua Medical School, Padua, Italy

## Abstract

**Objectives:**

To investigate the safety and clinical efficacy of tirofiban during primary percutaneous coronary interventions (pPCI).

**Background:**

Gp IIb/IIIa inhibitors (GPI) use during pPCI has declined over years, mainly for the increased hemorrhagic risk associated to their use and for the availability of potent, fast-acting oral antiplatelet drugs. However, several pharmacodynamic studies showed suboptimal platelet inhibition with P2Y12-blockers, such as prasugrel or ticagrelor.

**Methods:**

Patients with ST-segment elevation myocardial infarction (STEMI) undergoing pPCI were prospectively enrolled in a multicenter registry conducted in high-volume centers in Italy. All patients received intraprocedural tirofiban. The primary safety endpoint was the occurrence of in-hospital bleedings according to the Bleeding Academic Research Consortium definition. In-hospital major adverse coronary events (MACE, defined as death, reinfarction, stent thrombosis, and target vessel revascularization), final TIMI flow, myocardial blush grade, and ST-segment resolution were also evaluated.

**Results:**

A total of 472 patients (mean age 61 ± 11 years, 83% males) were enrolled in 16 Italian centers from October 2015 to June 2018. Mean basal thrombus grade score was 3.47 ± 1.25. PCI was performed by transradial approach in 88% of patients. We observed a very low rate of 30 days BARC bleedings (2.1%) and MACE (0.8%). Complete (>70%) ST-segment resolution was observed in 67% of patients.

**Conclusions:**

In the FASTER registry, the use of tirofiban during primary PCI, performed with a transradial approach in most cases, in patients with high thrombus burden was associated with high rates of complete ST-segment resolution and low rates of in-hospital bleeding and MACE.

## 1. Introduction

Patients with ST-segment elevation myocardial infarction (STEMI) have high expression of platelet P2Y12 receptors [1] and high platelet reactivity [2] that are associated to worse clinical outcome after revascularization with primary percutaneous coronary intervention (pPCI) [3]. Historically, these patients have been routinely treated with glycoprotein IIb/IIIa receptor inhibitors (GPI), based on their potent and fast-acting antiplatelet effect, which has been shown to reduce mortality in patients at high risk of thrombotic complications [4]. More recently, the results of some randomized controlled trials [5, 6], the growing awareness of the increased hemorrhagic risk associated with these drugs [7], and the availability of potent, fast-acting oral antiplatelet agents [8, 9] questioned and reduced their use in clinical practice [10]. Nevertheless, optimal levels of platelet inhibition during pPCI are unfrequently achieved after loading dose of either prasugrel or ticagrelor [11, 12], whereas the use of high-dose tirofiban on top of a loading dose of 600 mg clopidogrel was associated with improved myocardial reperfusion in the absence of increased bleedings in the On-TIME 2 trial [13]. Furthermore, transradial compared to transfemoral approach in patients with acute coronary syndrome undergoing PCI significantly reduced bleeding and improved survival [14].

We designed this prospective, multicenter registry in order to investigate the role of high-dose tirofiban on top of contemporary pharmacological therapy in a recent setting of STEMI patients undergoing pPCI.

## 2. Materials and Methods

The Favorite Approach to Safe and Effective Treatment for Early Reperfusion (FASTER) Registry is a multicenter observational registry promoted by the Italian Society of Interventional Cardiology (SICI-GISE) aiming to investigate safety and efficacy outcomes in patients with ST-elevation myocardial infarction and high thrombus burden undergoing primary PCI with high-bolus dose tirofiban. The study was supported by an unrestricted educational grant issued by Correvio International (Geneve, Switzerland). An external Clinical Research Organization (Clirest S.R.L., Ferrara, Italy) was responsible for data capture and management. The study was conducted in full conformity with the Declaration of Helsinki and Good Clinical Practice guidelines; the study protocol was approved by institutional review boards of participating centers, and written informed consent was obtained by each patient. The inclusion and exclusion criteria are given in Table 1. Coronary thrombus burden was visually assessed by the operator and graded from 0 (no angiographically visible thrombus) to 5 (thrombus which totally occludes the vessel) according to the TIMI group classification [15]. Vascular access site, oral antiplatelet therapy, and parenteral anticoagulant therapy were left to the discretion of the operator. A tirofiban bolus of 25 microgram/Kg given over a 3-minute period was administered to all patients, either pre-PCI or during PCI at operator's discretion; after the bolus, a continuous infusion at a rate of 0.15 microgram/Kg/min for up to 18 hours was strongly suggested, although not mandatory. In case of severe renal failure (creatinine clearance <30 ml/min), the dose of tirofiban was reduced by 50%. The primary safety endpoint was the occurrence of Bleeding Academic Research Consortium (BARC) bleedings during hospital stay. Secondary endpoints were major adverse coronary events (MACE) defined as all-cause death, reinfarction, stent thrombosis (according to Academic Research Consortium definition), and target vessel revascularization (TVR) during hospital stay. Reinfarction was defined as the occurrence of new symptoms and ECG signs associated with >20% increase in cardiac troponin I; stent thrombosis was defined according to Academic Research Consortium definition; TVR was defined as unplanned revascularization, either by PCI or CABG, of the vessel treated at the index PCI. The occurrence of both primary and secondary endpoints was also assessed at 30 days after PCI, either by phone interview or ambulatory visit. Data about TIMI flow and myocardial blush grade after PCI were collected. ST-segment resolution was assessed by comparing the sum of ST-segment elevation (∑ST) between ECG at presentation and 60 minutes after PCI; complete ST-segment resolution was defined as >70% reduction of ∑ST.

### 2.1. Statistical Analysis

Given the observational, descriptive nature of the study, no formal assessment of sample size was performed. Descriptive statistics (mean, median, interquartile range, minimum and maximum, and standard deviation) were calculated for continuous variables. Absolute frequencies and percentages were obtained for qualitative variables. *P* values less than 0.05 (2 sides) were considered statistically significant.

## 3. Results

**Figure 1:**
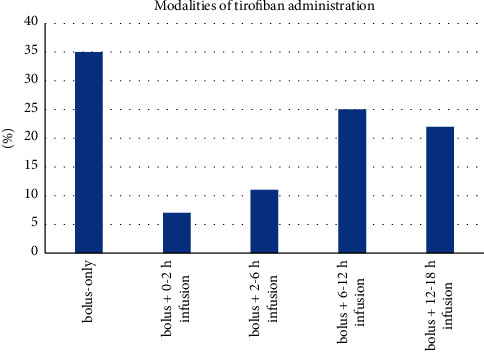
Patterns of administration of tirofiban in study patients.

A total of 472 patients (mean age 61 ± 11 years, 83% males) were enrolled in 16 Italian centers from October 2015 to June 2018. Clinical characteristics and blood test results at presentation are given in Table 2. STEMI was anterior in 46% of cases; the majority of patients were in Killip class 1 or 2. Procedural characteristics are given in Table 3. Most patients were treated within 6 hours from symptoms onset; radial access was largely prevalent (88%); a high thrombus burden was found in the majority of patients (mean TIMI thrombus grade score 3.47 ± 1.25). Thrombus aspiration was performed in about one-third of patients, balloon predilatation was performed in 71%, and at least 1 drug-eluting stent was implanted in 92% of patients. As far as antithrombotic therapy is concerned, 86% of patients were given aspirin and 73% a potent P2Y12 inhibitor (prasugrel or ticagrelor) before PCI; intravenous heparin was the only anticoagulant used (mean dose 5560 ± 2152 IU); no patient received bivalirudin or cangrelor. All patients received a periprocedural high-dose bolus of tirofiban, which was followed by up to 18 hours infusion in 65% of them. The length of infusion was variable: 0–2 hours in 7% of patients, 2–6 hours in 11%, 6–12 hours in 25%, and 12–18 hours in 22% (Figure 1). Final grade 3 TIMI flow and complete (>70%) ST-segment resolution were obtained in 90.5% and 67% of patients, respectively; pretreatment with either prasugrel or ticagrelor did not affect ST-segment resolution at univariate analysis (OR 1.01, 95% CI 0.67–1.51). Data about final myocardial blush were available in 239 patients, with grade 3 being reported in 49% of them. At discharge, 97% of patients received ASA, 8% clopidogrel, 30% prasugrel, and 62% ticagrelor.

The rate of in-hospital and 30-day adverse events is given in Table 4. In-hospital BARC bleedings were observed in 8 patients (1.7%) and major bleeding (BARC 3–5) in 4 (0.8%). As far as in-hospital MACEs are concerned, one patient died from refractory cardiogenic shock and 3 patients had definite stent thrombosis/reinfarction treated by new PCI.

## 4. Discussion

In this multicenter, observational registry, the use of high-bolus dose tirofiban in STEMI patients with high thrombus burden undergoing primary PCI was associated to a very low incidence of both major bleedings and MACE at 30 days. These results may be first related to the low clinical and procedural risk of the population enrolled. Indeed, patients were relatively young, mostly in Killip class I or 2, and on average, they presented with normal hemoglobin levels and renal function. Moreover, most of them were treated within 3 hours from symptoms onset, and pPCI was largely performed with transradial approach in high-volume centers. According to contemporary guidelines, most patients were discharged with double antiplatelet therapy with aspirin and prasugrel or ticagrelor, although pretreatment with these P2Y12 inhibitors was not associated with improved ST-segment resolution, similar to the findings of the ATLANTIC randomized trial [16]. As far as tirofiban is concerned, it is interesting to observe that a bolus-only strategy was adopted in about one-third of patients. Although no comparison can be made in our cohort between the bolus-only group and the bolus plus infusion group, given the low number of events, a bolus-only strategy coupled with early administration of potent oral P2Y12 inhibitors could represent an effective way to provide optimal platelet inhibition during pPCI, bridging the delay in the onset of antiplatelet activity of oral drugs and, at the same time, reducing the risk of bleeding complications associated with long-lasting GPI infusion. This strategy has been evaluated in the FABOLUS-PRO trial, a pharmacodynamic study which showed that the association of 60 mg loading dose of prasugrel with high-dose bolus of tirofiban achieved higher and consistent antiplatelet activity obviating the need of postbolus tirofiban infusion [17]. The possible benefit of this strategy was also assessed in an observational study showing that a bolus-only eptifibatide regimen was associated with similar infarct size but with significantly reduced major bleedings in STEMI patients undergoing pPCI as compared to conventional bolus and infusion treatment [18]. Another possible strategy to bridge the initial onset delay of oral P2Y12 inhibitors could be the administration of cangrelor, which is the only parenteral P2Y12 inhibitor and is characterized by a very fast onset of action [19]. However, data in STEMI patients from the CHAMPION program are limited, and no randomized comparison with prasugrel or ticagrelor is available [20]. Moreover, in a recent pharmacodynamic study in STEMI patients undergoing primary PCI, cangrelor yielded significantly inferior inhibition of platelet aggregation as compared to tirofiban, although both parenteral drugs were more effective than prasugrel, either administered as integral pills or chewed pills [21]. The latter study further supports a strategy of use of parenteral drugs (possibly GPI) to achieve immediate inhibition of platelet aggregation and to bridge the initial gap typical of orally administered drugs. Besides the antiplatelet effect, GPI was also shown to improve microvascular perfusion [22], whereas no data are available for cangrelor in this regard.

Our study presents several limitations. First of all, it is an observational study without a comparator arm. Second, despite the presence of high thrombotic burden at angiography, the population enrolled was at low risk, especially for bleeding; this may explain the low rate of MACE and bleeding events at 30-day follow-up which, in turn, precluded the possibility of subgroup analyses aiming to assess, if any, clinical and angiographic predictors of enhanced benefit of tirofiban on top of standard treatment. Third, most patients were treated by transradial approach, which is consistently associated with a striking reduction in access-related bleeding as compared to transfemoral approach in primary PCI [23], especially when GPI is used [24]. Fourth, pharmacodynamic evaluation of platelet aggregation was not performed. Finally, relevant angiographic data, such as number of diseased vessels, lesion length, and number of stents, are missing.

## 5. Conclusion

This study suggests that in STEMI patients undergoing transradial pPCI with high thrombus burden and low risk of bleeding, a strategy of bolus-only or bolus followed by short infusion of tirofiban on top of oral loading with potent P2Y12 inhibitors is associated with high rates of complete ST-segment resolution and low rates of both ischemic and hemorrhagic complications at 30 days. Adequately powered randomized controlled trials with clinical endpoints are needed in order to evaluate the safety and efficacy of the association of potent parenteral and potent oral antiplatelet drugs in STEMI patients undergoing pPCI.

## Figures and Tables

**Table 1:** Inclusion and exclusion criteria

Inclusion criteria	Exclusion criteria
Age 18–85 years	Contraindications to use of tirofiban
STEMI within 12 hours from symptoms onset with high thrombus burden	Patients with left bundle branch block
ST-segment elevation >1 mm in 2 adjacent ECG leads	Therapy resistant cardiogenic shock
Patients eligible for primary PCI within 120 minutes after first medical contact	Persistent severe hypertension (systolic BP >180 mmHg or diastolic BP >110 mmHg)
	Contraindication to anticoagulation
	Pregnant or breastfeeding women

**Table 2:** Clinical characteristics.

*n*	472
Age (years)	61.5 ± 11.2
Male gender (*n*, %)	391 (83)
Weight (Kg)	78.8 ± 14.6
History of smoking (*n*, %)	287 (61)
Diabetes mellitus (*n*, %)	79 (17)
Hypertension (*n*, %)	247 (53)
Dyslipidemia (*n*, %)	208 (45)
Previous myocardial infarction (*n*, %)	48 (10)
Previous TIA/stroke (*n*, %)	18 (4)
Previous PCI (*n*, %)	57 (12)
Previous CABG (*n*, %)	12 (3)
STEMI location (*n*, %)	
Anterior	217 (46)
Nonanterior	253 (54)
Killip class 3-4	12 (2.5)
Heart rate (bpm)	77 ± 18
Systolic blood pressure (mmHg)	134 ± 27
Hemoglobin (g/dl)	14.5 ± 2.5
Hematocrit (%)	42.5 ± 5.8
GFR (ml/min)	82.1 ± 23.5

**Table 3:** Procedural characteristics.

Time from symptom onset to PCI	
0–3 hours (*n*, %)	306 (65)
3–6 hours (*n*, %)	113 (24)
6–12 hours (*n*, %)	52 (11)
Radial access (*n*, %)	414 (88)
Basal TIMI flow (*n*, %)	
0	342 (72.5)
1	70 (14.8)
2	38 (8.1)
3	22 (4.7)
TIMI thrombus grade score	3.47 ± 1.25
Pretreatment with prasugrel or ticagrelor (*n*, %)	344 (73)
Tirofiban (*n*, %)	
Planned pre-PCI	374 (79)
Intraprocedural	98 (21)
Bolus-only	164 (35)
Bolus + infusion	308 (65)
Thrombus aspiration	
No	300 (64)
Manual	158 (33)
Rheolytic	14 (3)
Predilatation (*n*, %)	335 (71)
DES (*n*, %)	433 (92)
Final TIMI flow	
0	3 (0.6)
1	5 (1.1)
2	37 (7.8)
3	427 (90.5)
Final TIMI myocardial blush grade	
0	41 (17.2)
1	23 (9.6)
2	58 (24.3)
3	117 (49.0)
ST-segment resolution (*n*, %)	
<30%	28 (6)
30–70%	127 (27)
>70%	317 (67)

**Table 4:** In-hospital and 30 days adverse events.

	In-hospital	Discharge to 30 days	Overall
All BARC (*n*, %)	8 (1.7)	2 (0.4)	10 (2.1)
BARC 3–5 (*n*,%)	4 (0.8)	1 (0.2)	5 (1)
Death (*n*, %)	1 (0.2)	0 (0)	1 (0.2)
TVR (*n*, %)	1 (0.2)	0 (0)	1 (0.2)
Stent thrombosis (*n*, %)	3 (0.6)	0 (0)	3 (0.6)
IMA (*n*, %)	3 (0.6)	0 (0)	3 (0.6)

## Data Availability

The dataset used to support the findings of this study is available at the Contract Research Organization who followed the study: Clirest S.R.L. Via Valdicuore, 17, 44124 Ferrara, Italy.
